# Management of Hypoparathyroidism

**DOI:** 10.1002/jbmr.4716

**Published:** 2022-10-31

**Authors:** Aliya A. Khan, Gordon Guyatt, Dalal S. Ali, John P. Bilezikian, Michael T. Collins, Karel Dandurand, Michael Mannstadt, Deborah Murphy, Iman M’Hiri, Mishaela R. Rubin, Robert Sanders, Muhammad Shrayyef, Heide Siggelkow, Gaia Tabacco, Yu-Kwang Donovan Tay, Stan Van Uum, Tamara Vokes, Karen K. Winer, Liang Yao, Lars Rejnmark

**Affiliations:** 1 Division of Endocrinology and Metabolism, McMaster University, Hamilton, Canada; 2 Department of Health Research Methods, Evidence and Impact, McMaster University, Hamilton, Canada; 3 Vagelos College of Physicians and Surgeons, Columbia University, New York, NY, USA; 4 National Institute of Dental and Craniofacial Research, National Institutes of Health, Bethesda, MD, USA; 5 Endocrine Unit, Massachusetts General Hospital and Harvard Medical School, Boston, MA, USA; 6 Hypoparathyroidism Association. Inc., Lemoore, CA, USA; 7 Bone Research and Education Centre, Oakville, Canada; 8 Department of Medicine, University of Toronto, Canada; 9 Clinic of Gastroenterology, Gastrointestinal Oncology and Endocrinology, University Medical Center Goettingen, Goettingen, Germany; 10 MVZ Endokrinologikum Goettingen, Goettingen, Germany; 11 Unit of Metabolic Bone and Thyroid Diseases, Fondazione Policlinico Universitario Campus Bio-Medico, Rome, Italy; 12 Unit of Endocrinology and Diabetes, Campus Bio-Medico University of Rome, Rome, Italy; 13 Department of Medicine, Sengkang General Hospital, Singhealth and Duke-NUS Medical School, Singapore, Singapore; 14 Department of Medicine, Western University, London, Canada; 15 Department of Medicine, University of Chicago, Chicago, IL, USA; 16 Eunice Kennedy Shriver National Institute of Child Health and Human Development, National Institutes of Health, Bethesda, MD, USA; 17 Department of Endocrinology and Internal Medicine, Aarhus University Hospital, Aarhus, Denmark

**Keywords:** PARATHYROID-RELATED DISORDERS, DISORDERS OF CALCIUM/PHOSPHATE METABOLISM, CELL/TISSUE SIGNALING, ENDOCRINE PATHWAYS

## Abstract

Hypoparathyroidism (HypoPT) is a rare disorder characterized by hypocalcemia in the presence of a low or inappropriately normal parathyroid hormone level. HypoPT is most commonly seen after neck surgery, which accounts for approximately 75% of cases, whereas approximately 25% have HypoPT due to nonsurgical causes. In both groups of patients, conventional therapy includes calcium and active vitamin D analogue therapy aiming to maintain serum calcium concentration in the low normal or just below the normal reference range and normalize serum phosphorus, magnesium concentrations, and urine calcium levels. The limitations of conventional therapy include wide fluctuations in serum calcium, high pill burden, poor quality of life, and renal complications. Parathyroid hormone (PTH) replacement therapy may improve the biochemical profile in those in whom conventional therapy proves unsatisfactory. Based on a systematic review and meta-analysis of the literature, the panel made a graded recommendation suggesting conventional therapy as first line therapy rather than administration of PTH (weak recommendation, low quality evidence). When conventional therapy is deemed unsatisfactory, the panel considers use of PTH. Because pregnancy and lactation are associated with changes in calcium homeostasis, close monitoring is required during these periods with appropriate adjustment of calcium and active vitamin D analogue therapy to ensure that serum calcium remains in the mid to low normal reference range in order to avoid maternal and fetal complications. Emerging therapies include molecules with prolonged PTH action as well as different mechanisms of action that may significantly enhance drug efficacy and safety.

## Graded Panel Recommendation

1. In patients with chronic hypoparathyroidism (HypoPT), the panel suggests conventional therapy as first line therapy rather than administration of parathyroid hormone (PTH) (weak recommendation, low quality evidence).

Comment - When conventional therapy is deemed unsatisfactory the panel considers use of PTH.

## Ungraded Panel Recommendations

In patients with HypoPT, the panel proposes:

1.1. Treat with calcium and active vitamin D analogue therapy, with a goal to raise serum calcium (albumin adjusted or ionized) into the target range; ie, the lower half of the normal reference range or just below the normal reference range. At this time, it is not clear how to best balance the doses of calcium and active vitamin D analogue therapy.

1.2. Alleviate symptomatic hypocalcemia while avoiding hypercalcemia.

1.3. Avoid hypercalciuria when titrating calcium and active vitamin D analogue therapy, aiming for low normal plasma calcium levels.

The panel proposes achieving a 24-hour urinary calcium level <6.25/7.5 mmol/24 hours (250/300 mg/24 hours) for adult women and men, respectively. Data from the general population has shown a relationship between hypercalciuria and the development of renal stones—such data does not exist in patients with HypoPT; however, panel members infer that hypercalciuria may be associated with a higher risk of renal stones in patients with HypoPT as well and thus seek to avoid hypercalciuria.

1.4. Avoid hyperphosphatemia. Panel members prescribe calcium supplements with meals to serve as phosphate binders, implement a low phosphate diet in adults and judiciously use active vitamin D analogue therapy. No data are available on the use of other types of phosphate binders in HypoPT.

Hyperphosphatemia may be associated with an increased incidence of ectopic calcification; however, currently there is no evidence of this in HypoPT.

1.5. Treat to normalize plasma magnesium levels. Magnesium supplements can be used as tolerated by the patient.

1.6. Aim to achieve a 25-hydroxyvitamin D level in the normal reference range.

1.7. Consider treating hypercalciuria with thiazide diuretics in conjunction with a low sodium diet with careful monitoring of serum magnesium, potassium, and renal function.

1.8. Consider PTH replacement therapy in patients who are not adequately controlled on conventional therapy. Inadequate control is considered to be any one of the following: (i) symptomatic hypocalcemia; (ii) hyperphosphatemia; (iii) renal insufficiency; (iv) hypercalciuria; (v) poor quality of life.

1.9. Individuals with poor compliance, malabsorption or who are intolerant of large doses of calcium and active vitamin D may also benefit from PTH therapy.

## Ungraded Consensus Panel Recommendations During Pregnancy and Lactation

In pregnant women with HypoPT, the panel proposes the following:

2.1. Aim to achieve serum calcium (albumin adjusted or ionized) in the mid to low normal reference range throughout pregnancy.

2.2. Aim to achieve serum phosphorus, magnesium and 25-hydroxyvitamin D levels in the normal reference range.

2.3. Adjust doses of calcium and active vitamin D according to serum calcium (albumin adjusted or ionized) every 3–4 weeks during pregnancy and lactation, with increased frequency in the month preceding and following parturition as well as in the presence of symptoms of hypercalcemia or hypocalcemia.

2.4. Work closely with the obstetrician and pediatrician to optimize pregnancy outcomes.

2.5. Avoid using thiazide diuretics and parathyroid hormone and parathyroid hormone analogues during pregnancy.

These recommendations are intended to guide practice and are not intended to be used for the development of reimbursement policies.

## Introduction

Hypoparathyroidism (HypoPT) is a rare condition characterized by hypocalcemia in the presence of a low or inappropriately normal serum parathyroid hormone (PTH).^(1-4)^ It is associated with significant complications and symptoms (Table 1). HypoPT is most commonly caused by removal of or damage to the parathyroid glands or their blood supply during neck surgery. Approximately 25% of cases are nonsurgical, most commonly due to autoimmune disease or genetic mutations.^(1-4)^ Rarely, magnesium abnormalities, infiltrative causes, radiation, or metastatic disease may result in HypoPT. Hypocalcemia may also occur due to PTH resistance (so called pseudohypoparathyroidism), which is not covered by these guidelines.

In this manuscript we present an evidence-based approach to the management of HypoPT. Areas with very low-quality evidence are addressed by descriptions of expert panel practice for patients with HypoPT.

## Methodology

Two systematic reviews informed the recommendations provided in this document: a survey of panel practice^(5)^ and a review of randomized trials of PTH versus conventional therapy in patients with HypoPT.^(6)^

The reviews utilized methods designed to produce trustworthy results including a structured clinical questionnaire; a comprehensive search of relevant literature; duplicate assessment of eligibility, risk of bias, and data abstraction; and, for the randomized trial review, application of Grading of Recommendations Assessment, Development and Evaluation (GRADE) methodology to rate the quality of evidence.

This document includes two types of recommendations: graded and ungraded. Graded recommendations^(7)^ followed a structured process that included framing questions in patient/intervention/comparator/outcome format^(8)^; conduct of a systematic evidence search and associated summary as described in the previous paragraph^(9)^; specification of values and preferences^(10)^; and classifying and presenting recommendations as strong or weak with the corresponding quality of evidence.^(11)^ Ungraded recommendations involved none of these structured approaches and are presented as descriptions of the practice of the panelists in managing patients with HypoPT. Ungraded recommendations are presented as “we propose.” The intent was to achieve consensus on all recommendations. There was no provision for voting.

Because there is almost no published evidence directly addressing the frequency of monitoring, the panel planned and conducted a survey of 97 international experts in parathyroid disease of whom 70 responded. This data was analyzed and discussed over a series of meetings. Panel experts were asked how they treat and monitor their patients with HypoPT in order to maintain optimal quality of life (QoL) and avoid complications. The panel carefully reviewed the practices and set a threshold requiring 70% of respondents to adopt this monitoring practice in at least 70% of their patients before incorporating this practice into a practice recommendation. The survey is published in a separate article^(5)^ with main findings on therapies summarized in this work.

## Conventional Therapy

In addition to a calcium-rich diet, the conventional therapy for patients with HypoPT includes calcium supplementation and active vitamin D analogue therapy (calcitriol, alfacalcidol, or dihydrotachysterol) in varying doses.^(1-4)^ Doses are titrated to obtain serum calcium levels in the lower half or slightly below the reference interval.

Oral calcium preparations contain varying amounts of elemental calcium: calcium carbonate (40% elemental calcium), calcium citrate (21%), calcium gluconate (9%), and calcium lactate (13%). Calcium salts are absorbed well^(12,13)^ and also act as phosphate binders to lower serum phosphorus when given with meals.^(13)^ If calcium carbonate is used, it should be taken with a meal for enhanced absorption. The absorption of calcium in the form of calcium citrate is not affected by food intake.^(14)^

The limitations of current conventional therapy include a significant pill burden with complicated regimens, fluctuations in serum calcium, and diminished QoL. Active vitamin D increases the absorption of calcium and phosphorus in the small bowel.^(15)^ This increases serum calcium and can also result in increased renal filtered calcium load with further elevations in urinary calcium as well as elevations in serum phosphorus. Hypercalciuria and renal calcification appear to be common complications of long-term conventional therapy. Currently, however, we have not been able to clearly document a correlation between hypercalciuria and the development of kidney stones or renal impairment in patients with HypoPT.^(16-18)^

It is recommended to monitor serum phosphorus and urine calcium and aim to normalize both of these parameters in patients on conventional therapy.^(1,5)^ This may require reductions in the doses of calcium and active vitamin D.^(19)^

Patients with nonsurgical HypoPT due to an activating mutation in the calcium sensing receptor (ie, autosomal dominant hypocalcemia [ADH]) are more likely to develop hypercalciuria in response to calcium supplements.^(20)^

## PTH Therapy

Replacement therapy with intact recombinant human PTH (1-84) [rhPTH (1-84)] has been approved as an adjunct to conventional therapy by regulatory agencies. In addition, effects of therapy with PTH (1-34) in HypoPT in comparison to conventional therapy has been investigated in several studies in adults and children (Table 2).

### PTH (1-34)

A series of studies by Winer and colleagues^(21)^ demonstrated that synthetic human PTH (1-34) (hPTH) given once or twice daily, maintained eucalcemia, reduced urine calcium excretion, and increased phosphorus excretion. The effects of PTH (1-34) injections on mineral metabolism differed according to the etiology of HypoPT. Winer and colleagues^(22,23)^ reported in an open-label, randomized, crossover trial that twice-daily, compared to once-daily PTH (1-34) injections, maintained serum calcium in the near-normal range over 24 hours and significantly reduced the total daily PTH dose from that required under the once-daily regimen. Synthetic hPTH (1-34) was also safe and effective over a 3-year period in both adults and children.^(24,25)^ Furthermore, children treated with hPTH (1-34) in a 3-year randomized parallel trial maintained normal linear growth, weight gain, and renal function with no difference in lumbar spine and whole-body bone mineral density (BMD) *Z*-scores compared to children receiving conventional therapy. PTH was associated with elevated bone turnover markers compared to conventional therapy.^(24,25)^ Over 10 years, Winer and colleagues^(26)^ reported that children with autoimmune polyendocrine syndrome type 1 (APS-1) or ADH1 treated with PTH (1-34) injections had normal height velocity and bone mineral accretion velocities. The baseline evaluation of nonsurgical HypoPT patients receiving calcitriol and calcium revealed a high prevalence of renal insufficiency or renal calcification.^(25-27)^ There was no change in creatinine clearance for the study duration in the long-term studies of the effects of treatment with hPTH.^(25,26)^ Synthetic hPTH (1-34) has been associated, in one study, with hypocitraturia, a risk factor for renal calcification.^(28)^

A more physiologic approach, by Winer and colleagues,^(29,30)^ involves continuous delivery of hPTH (1-34) by subcutaneous infusion pump. In a randomized cross-over study comparing hPTH (1-34) delivered by an infusion pump vs twice-daily injections, patients receiving continuous pump delivery manifested normalization of serum calcium with less fluctuation in serum calcium, phosphorus, and magnesium and reduced urine calcium with normalization of bone turnover markers. Daily dose of hPTH (1-34) and magnesium requirements were also significantly reduced with infusion pump delivery of hPTH in comparison to twice daily hPTH (1-34) injections.

Although synthetic hPTH (1-34) is not clinically available, these findings have been replicated with the clinically available recombinant human PTH (1-34) (rhPTH) in adults,^(31)^ children,^(32-36)^ and in infants with refractory, life-threatening HypoPT.^(37-39)^

### PTH (1-84)

Full-length rhPTH (1-84) has been evaluated in placebo-controlled and open-label studies.^(40-43)^ The REPLACE study was a 24-week, double-blind, placebo-controlled, phase 3 study conducted in 134 patients randomized to rhPTH (1-84) or placebo. The primary end point (≥50% reduction in calcium and calcitriol doses with maintenance of normal serum calcium) was met in 53% of patients receiving rhPTH (1-84) versus 2% of patients receiving placebo.^(41)^ The decreased need for calcium and active vitamin D supplementation was also observed in both the dose-adjusted^(40)^ (Columbia University) and fixed dose (100 μg/day, Aarhus University)^(42)^ studies. In the randomized controlled trials (RCTs) of rhPTH (1-84), there was no statistically significant difference in urinary calcium excretion between rhPTH and conventional therapy^(41,42)^; however, in the open-label extension of REPLACE, mean urinary calcium level declined into the normal range,^(43)^ similar to what was seen in an 8-year open-label study.^(40)^ Serum phosphorus and the calcium/phosphorus product decreased in the REPLACE and extension study,^(5,6,41,43)^ findings not replicated in the fixed-dose or adjusted-dose studies.^(40,42)^

Renal function was found to be stable in the rhPTH (1-84) studies.^(40-43)^ Despite normalization of urine calcium, renal calcifications were not eliminated as nephrolithiasis was still reported in the REPLACE extension study^(43)^ and in the Columbia University study,^(40)^ possibly due to hypocitraturia, but this was not assessed. Hypercalcemia, at various times during the studies, has been observed: 18% in the REPLACE study,^(41)^ 34% in the fixed-dose study,^(7,42)^ and 30% in the Columbia University studies.^(40,44)^ Hypocalcemia was also observed in the RCTs: 38% in the study drug arm versus 23% in placebo in the REPLACE study,^(41)^ 29% in the PTH arm and 53% in the placebo arm in the Aarhus University study,^(42)^ and 13%, with one hospitalization, in the Columbia University study.^(8,40)^

Effects on bone turnover markers, bone density, and dynamic and structural changes at the tissue level with rhPTH (1-84) are similar to the effects seen with PTH (1-34).^(29,30,32)^ In general, there is an initial marked increase in bone turnover markers, which subsequently decreases and plateaus with long-term treatment. The new steady state is higher than baseline but within the normal reference range. Bone density is in general stable but with decreases observed at the 1/3 radial site. By dynamic histomorphometry as evaluated by bone biopsy, and by high-resolution peripheral quantitative computed tomography (HRpQCT)^(9,45)^ an increase in cortical porosity has been observed.^(42-44)^ The clinical implications of increased cortical porosity are not known, and fracture data are not available.

In the United States, rhPTH (1-84) was approved in 2015 as a once daily subcutaneous administration as an adjunctive treatment for adults with HypoPT not well controlled on conventional therapy.^(46)^ Notably, after discussions with the US Food and Drug Administration, rhPTH was recalled in the United States in September 2019. This was reported to be due to an issue related to rubber particulates originating from the rubber septum of the cartridge.^(47)^ In Europe, rhPTH (1-84) has not been recalled and is approved as add-on therapy to treatment with calcium and active vitamin D supplements when these treatments have been inadequate in the care of HypoPT.

## PTH Therapy in Comparison to Conventional Therapy

A systematic review and meta-analysis of the literature from inception to May 2022 was conducted to evaluate the benefits and harms of PTH therapy in comparison to conventional therapy in managing patients with chronic HypoPT.^(6)^ Seven studies met the eligibility criteria listed in Table 2. The studies were small, however, and did not report on the eight complications identified as being associated with HypoPT. The studies demonstrate that PTH therapy may enable a larger number of patients to reduce the dose of calcium and active vitamin D by 50% or more. Reductions in serum phosphorus and increases in episodes of hypercalcemia were found to be statistically significant effects of PTH therapy in comparison to conventional therapy. PTH therapy was associated with an increase in bone remodeling reflected by an increase in the biomarkers alkaline phosphatase, osteocalcin, and urine pyridinoline. Serious adverse effects were very infrequent. In the meta-analysis PTH therapy was associated with small improvements in QoL and reduction in pill burden.^(6)^ Because the studies had a very small sample size it was not possible to appreciate the benefits and risks of PTH therapy on patient important outcomes Tables 2 and 3.

Although studies with PTH (1-34) did not report improvements in QoL^(23,29,48)^ drug efficacy is likely to be affected by the dose and mode of administration as well as the half life of the PTH molecule. Studies with PTH (1-84) therapy demonstrated significant improvements in QoL^(49)^ and this was also observed with the TransCon PTH molecule which has a 60-hour half-life and is currently in phase 3 trials.^(50)^

## HypoPT in Pregnancy

**Fig. 1.**
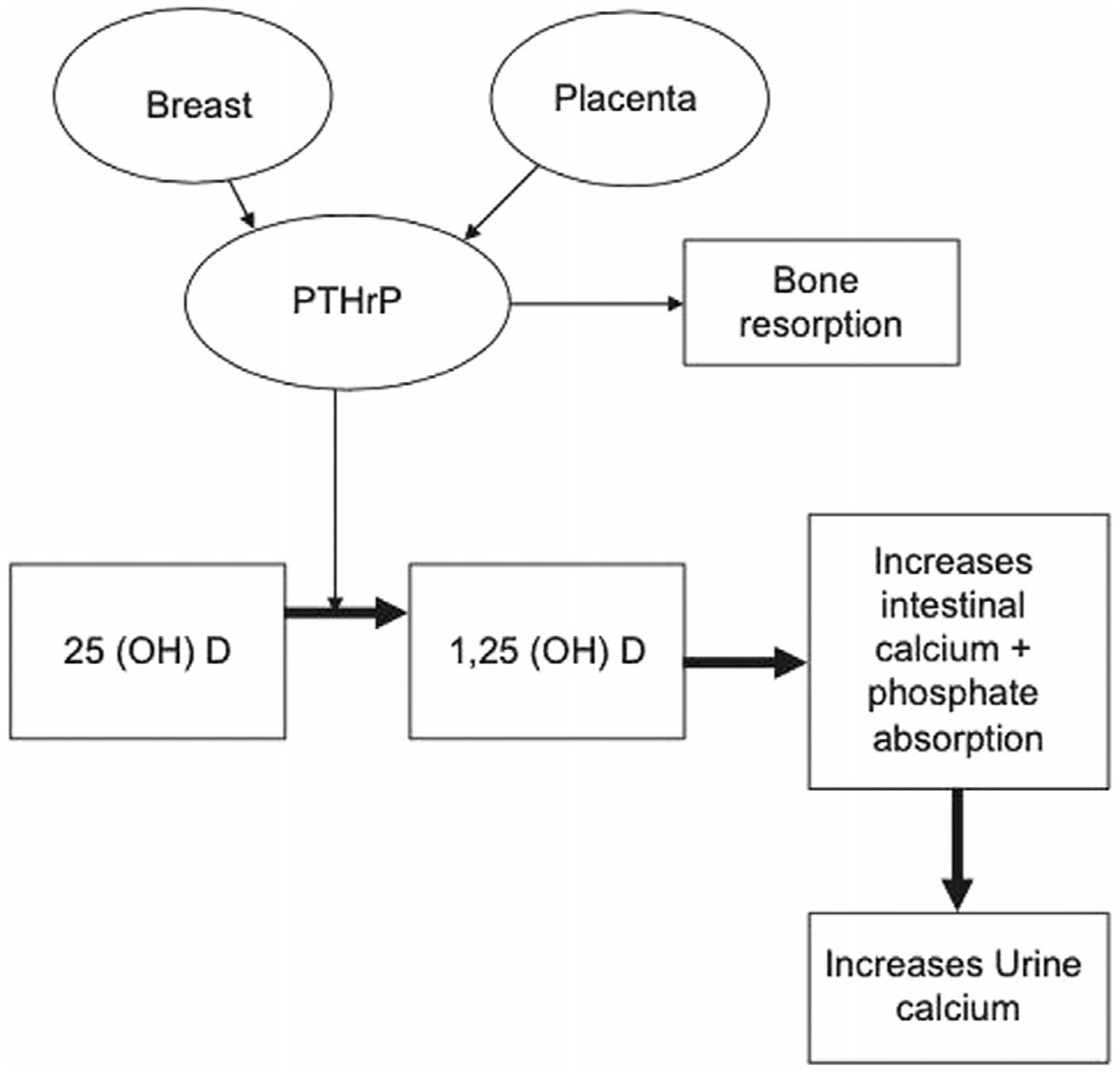
Maternal effects of PTHrP during pregnancy. Reproduced with permission from Khan and colleagues.^(51)^

Pregnancy is associated with a significant change in calcium homeostasis (Fig. 1). These changes result in altered requirements for calcium and active vitamin D analogue.^(51)^

Ionized calcium and albumin-adjusted calcium remain normal during pregnancy in women with normal parathyroid function; however, total serum calcium may decline due to the increase in intravascular volume. Therefore, monitoring parathyroid function requires evaluating albumin-adjusted or ionized calcium.^(52-54)^ Serum phosphorus and 25(OH)D_3_ levels are not affected by pregnancy.^(52-55)^ Endogenous synthesis of 1,25 (OH)_2_-D3 increases twofold to threefold as early as the first trimester, resulting in enhanced intestinal calcium absorption (Fig. 1). The enhanced synthesis of 1,25(OH)_2_D_3_ is at least in part caused by increased production of PTH-related protein (PTHrP) by the placenta and breast tissue independent of whether the parathyroid glands are functioning. Enhanced maternal intestinal calcium absorption enables increased transfer of calcium from the mother to the fetus and ensures fetal calcium requirements are met. In women with (residual) parathyroid function, this will cause suppression of PTH.^(53-61)^ Hypercalciuria may increase during pregnancy with increased renal filtered calcium load. Data on risk of renal calcification in HypoPT during pregnancy are lacking.

In general, pregnant women with HypoPT are treated similar to nonpregnant women and calcium and active vitamin D analogues are safe. However, it should be recognized that the increased endogenous synthesis of 1,25(OH)_2_D_3_ during pregnancy, which does not require PTH, may reduce the requirements for exogenous active vitamin D analogue and calcium supplements. However, this may be countervailed by increases in estimated glomerular filtration rate (eGFR) and increased urinary calcium losses, and transfer of calcium to the fetus that may necessitate increases in the dose of the supplements. It is difficult to predict whether an increase ora decrease in the doses of calcium and active vitamin D are needed. A recent case series reported that although average doses of active vitamin D analogue, and calcium supplements did not change during pregnancies, more than 20% increase or decrease in the dose of active vitamin D analogue was required in more than one-half of the patients to maintain normocalcemia.^(62)^ This is in agreement with several case reports suggesting that the doses of calcium and active vitamin D analogue required during pregnancy increased.^(63-66)^ An additional reason for increased active vitamin D analogue requirement is variation in dietary calcium intake. Many of the case reports describing the need for higher supplementation of active vitamin D analogue did not report if the patient was taking calcium or if the dose of calcium being administered changed.^(55,56,59-62)^

Inadequate calcium intake in the first trimester may impact skeletal development in the last trimester, the period of greatest bone accrual in the developing fetal skeleton (80%).^(67,68)^ Further, maternal hypercalcemia can result in fetal parathyroid gland suppression and hypocalcemia in the neonate.^(69)^ The risk of maternal hypercalcemia is highest shortly before and after giving birth, probably due to increased synthesis of PTHrP during this period.^(62)^ Conversely, maternal hypocalcemia is also harmful because it may lead to secondary hyperparathyroidism in the fetus.^(10-13,70-73)^ This in turn can result in demineralization of the fetal skeleton with the possibility of fractures in utero. Maternal hypocalcemia can also result in uterine contractions and is associated with an increased risk of preterm labor or miscarriages.^(74)^ Taken together, perinatal complications are increased in women with HypoPT, confirmed by a recent case series that reported caesarean delivery in 59% of pregnant women with HypoPT, with 24% being performed as an emergency. Moreover, 24% of the pregnancies were complicated by polyhydramnios, dystocia, and/or perinatal hypoxia.^(62)^

Calcium, vitamin D, and active vitamin D analogue can safely be continued during pregnancy. There is inadequate safety data on the use of thiazide diuretics and PTH during pregnancy (risk category B and C, respectively) and they should be discontinued as early as possible in pregnancy.^(75)^ Monitoring serum calcium, phosphorus, magnesium, and renal function every 3–4 weeks during pregnancy and with an increase to once weekly during the last months of pregnancy and the first few months after birth is advisable. Of importance, breastfeeding causes increased synthesis of PTHrP, which may influence needs for calcium supplements and active vitamin D. Accordingly, close attention has to be given to carefully modifying dose adjustments both when starting and stopping lactation.

The doses of calcium and active vitamin D analogue may need to be adjusted to maintain albumin-adjusted or ionized serum calcium in the low to mid normal reference range. Ensuring optimal response to modifications likely requires monitoring serum calcium levels every 2–3 days until stable.

## Pediatric Perspective

Compared to adults with surgical HypoPT, children with nonsurgical HypoPT present greater management challenges and manifest a relatively high prevalence of kidney and brain calcifications when treated with conventional therapy.^(14,15,21,26)^ In such children, large doses of calcium and active vitamin D are often not practical or effective. These findings have prompted studies with hPTH (1-34) given as twice daily injections or delivered by pump with good responses without evidence of harm.^(14,16,26,30)^ Reports of individualized dosing of subcutaneous hPTH (1-34) in children have suggested that smaller, more frequent doses avoid night-time hypocalcemia and hypomagnesemia while maintaining normal urine calcium concentrations.^(17,23)^ Mineral homeostasis improved further when hPTH (1-34) was delivered by an insulin pump.^(30)^

Studies demonstrating improved metabolic control including reduction in urine calcium with rhPTH (1-34) in genetic forms of HypoPT found effective doses ranged from 0.3 to 0.8 μg/kg/day given in two divided doses.^(34,35)^ When delivered by an insulin pump, total daily rhPTH (1-34) doses were substantially lower (0.1–0.5 μg/kg/day) compared to injection doses when studied in children with HypoPT. In these reports, rhPTH (1-34) delivered by pump maintained long-term serum calcium, phosphorus, and urinary calcium levels in the normal range with substantially reduced incidence of adverse events and hospitalizations.^(32,33)^

Management of HypoPT in infants and toddlers is described in a few case reports of emergency treatment of hypocalcemia,^(37,38)^ long-term PTH (1-34) therapy in a premature infant with DiGeorge,^(76)^ or conventional therapy in two 2-month-old infants with GCMB2 mutations and a 6-month-old with Sanjad Sakati syndrome where intercurrent illness required escalation of vitamin D and calcium doses.^(77)^ Genetic forms of HypoPT rarely manifest as neonatal hypocalcemia, which is more likely associated with prematurity, birth asphyxia, maternal diabetes, hypomagnesemia, metabolic alkalosis, increased phosphate load, or defects in vitamin D metabolism.^(78,79)^ Maternal hyperparathyroidism may lead to functionally immature parathyroids in the neonate and usually requires treatment with calcium and vitamin D analogs in pharmacologic doses. Newfield^(38)^ described a 17-day-old infant with HypoPT who received short-term rhPTH (1-34) therapy during a hypocalcemic crisis associated with seizures. After 2 days of treatment with intravenous calcium and oral calcitriol, a single 1 μg/kg subcutaneous rhPTH (1-34) injection raised the serum calcium to the normal range within 4 hours. Another case report in older children^(39)^ described short-term use of rhPTH (1-34) for management of a hypocalcemic crisis. Mishra and colleagues^(39)^ described a 10-year-old girl with APS-1 and a 12-year-old girl with postsurgical HypoPT with refractory hypocalcemia despite multiple calcium gluconate infusions and high doses of calcitriol. Subcutaneous rhPTH (1-34) injections normalized calcium levels within 48 hours in both patients.

Children with HypoPT require adequate dietary calcium, magnesium, and vitamin D, in order to support normal linear growth and bone accrual.^(80)^ A phosphate restricted diet may be inadvisable in children with HypoPT as children will be required to reduce dairy intake.^(81,82)^ Most pediatric patients and their families welcome nutrition counseling in the early stages of the disease process.

## Emerging Therapies for HypoPT

There are at least five new therapies on the horizon for treating HypoPT. Four involve targeting the parathyroid hormone receptor (PTH1R), and one targeting the calcium-sensing receptor (CaSR).

TransCon PTH (TC PTH), an inactive prodrug in which PTH (1-34) is linked to polyethylene glycol, provides stable PTH levels with an effective half-life of approximately 60 hours, is the farthest along in development. In preclinical and a phase 1 study, PTH levels were within the physiologic range for 24 hours at steady state.^(83,84)^ In a 4-week, blinded phase 2 trial, daily TC PTH was compared to conventional therapy in 59 adults with chronic HypoPT. This was followed by a 26-week open-label extension period during which patients were titrated to achieve a low normal serum calcium. The primary efficacy composite endpoint at the end of the 4-week blinded period included a normal serum calcium, and a fractional excretion of calcium (FECA) either within normal range or reduced by 50%, discontinuation of all active vitamin D analogue, supplements, and a reduction of calcium supplementation to ≤1000 mg/day (or ≤500 mg/day; secondary endpoint), inclusive. Eighty-two percent of the TC PTH-treated patients achieved independence from conventional therapy at week 4, as compared to only 15% of patients treated with placebo. At baseline, participant scores on the validated Short Form 36 Health Survey (SF-36) tool were below average, and by week 4 reached the normal range on both the SF-36 and the HypoPT patient experience scale (HPES). TC PTH was well-tolerated and there were no adverse events of hypocalcemia or hypercalcemia requiring a visit to hospital, emergency room, or urgent care. In accordance with the well-known effects of PTH, data from the first 6 months of treatment show an increase in bone turnover with a decrease in BMD.^(85)^ In the phase 3 RCT of TC PTH 18 μg daily or placebo both coadministered with conventional therapy, 26-week data demonstrated maintenance of eucalcemia. At week 26, 93% (57/61) of participants treated with TC PTH achieved independence from conventional therapy. Treatment with TC PTH over 26 weeks improved QoL and also normalized mean 24-hour urine calcium. TC PTH was well tolerated with mild or moderate adverse events. No study drug–related withdrawals occurred.^(86)^

An injectable long-acting PTH analogue (LA-PTH), a hybrid molecule with both PTH and PTHrP homology, increased serum calcium and reduced serum phosphorus in a thyroparathyroidectomized rat model of HypoPT and had prolonged effects on serum calcium in monkeys.^(87,88)^ Phase-1 trial data with this drug, AZP-3601, has been presented.^(89)^

Another PTHR1-directed approach has been the development of an orally active, small molecule agonist of the PTHR1, PCO371.^(90)^ It increases serum calcium and decreases serum phosphorus in thyroparathyroidectomized rats.^(91)^ This study has been terminated due to increases in liver enzymes in humans. (AAK, personal communication with Chugai).

A novel oral PTH molecule is being studied for the treatment of HypoPT (NCT02152228). PTH (1-34) is complexed with the excipients salcaprozate sodium and soybean trypsin inhibitor to both facilitate absorption across the gastrointestinal wall while at the same time protect the peptide from proteolysis. Results of a 16-week, open-label pilot study showed a 42% reduction in supplemental calcium, median serum calcium levels remained above the lower target of (>7.5 mg/dL); serum phosphorus levels were within the normal range throughout the study. 24-hour urine calcium was lowered on therapy in comparison to the baseline value; however, this difference was not significant.^(92)^

Antagonists of the CaSR are another novel approach to promote PTH secretion in patients with intact parathyroid glands and increase renal tubule reabsorption of calcium, with the net effect of raising serum calcium.^(93)^ This may be the ideal treatment for patients with HypoPT due to gain-of-function mutations of the CaSR, ADH1. This concept is supported by the results of a proof-of-concept trial with the calcilytic, NPSP795, which demonstrated a dose-dependent and study drug concentration-dependent increase in PTH in patients with ADH1.^(94)^ Early results from Period 1 of a Phase 2 trial in which six patients with ADH1 with four distinct CASR genotypes were treated with escalating doses of the calcilytic encaleret (NCT04581629) showed that, serum calcium, phosphorus, and magnesium were largely normalized and maintained within the normal range by day 5 while off of all supplements. Urinary calcium excretion, which was elevated at baseline, became normal in three patients and undetectable in three while on encaleret.^(95)^

## Patient Perspective

Members of The HypoPARAthyroidism Association, Inc. believe that a single treatment strategy may not be ideal for all patients. Each patient with HypoPT has different symptoms and complications. Patients think of each person like a snowflake; no two are alike. Many patients feel that conventional therapy keeps them alive, but with poor QoL and at the cost of long-term consequences, including advanced renal disease. Patients often report that their doctors do not understand how to diagnose and treat HypoPT, and frequently minimize or dismiss their symptoms. Great hope was raised when PTH (1-84) was approved, and while it was not ideal for all patients it was progress. Treatment options that will address the underlying cause of HypoPT and improve QoL are needed. The HypoPARAthyroidism Association fully supports the plan of the task force to improve treatment and quality and continuity of care for patients with HypoPT globally.

## Summary

Active vitamin D analogue therapy, in combination with calcium supplements, remains the cornerstone for the treatment of HypoPT. The primary aims are to normalize serum calcium while keeping patients symptom free. Despite careful titration and frequent monitoring of pharmacologic therapy, treatment is associated with a high pill burden, fluctuations in serum calcium and other biochemical abnormalities (eg, hyperphosphatemia, hypercalciuria) in addition to a number of complications, including reduced QoL and kidney disease.

Women with HypoPT who become pregnant are at an increased risk of unpredictable fluctuations in serum calcium as well as adverse pregnancy outcomes and require more frequent monitoring.

Replacement therapy with PTH has been evaluated in randomized and in observational trials. The results are weakened in some cases by experimental design and small sample sizes that result in wide confidence intervals. Although the resulting evidence is of low quality, PTH replacement is nevertheless a valuable option for patients who cannot be adequately controlled on conventional therapy and is an important step forward. More physiologic approaches to PTH replacement with smaller, more frequent doses of PTH, long-acting PTH-analogues, or by continuous PTH infusion may offer an essential alternative to conventional therapy for many patients with HypoPT.

The panel suggests, therefore, conventional therapy rather than PTH therapy (weak recommendation, low quality evidence), as the initial approach to all patients with HypoPT, but adds that PTH should be considered if conventional therapy is deemed unsatisfactory (ungraded comment). New molecules with prolonged PTH action, PTH analogues, PTH receptor agonists, and calcilytic molecules are under development and are promising for the future treatment of HypoPT.

## Research Agenda

The following areas of research are expected to improve the quality of care available for HypoPT:

1. Adequately powered, long-term, prospective controlled studies evaluating PTH versus conventional therapy on important patient outcomes.

2. Prospective controlled studies of the determinants of nephrocalcinosis and renal insufficiency in patients with HypoPT.

3. Prospective studies in HypoPT during pregnancy evaluating optimal management strategies.

4. Controlled, long-term studies evaluating the effects of PTH 1-34 and other PTH molecules delivered by pump.

5. Studies to evaluate the long-term effects of PTH therapy on bone quality and strength.

6. Controlled studies on phosphate restriction in children with severe hyperphosphatemia: Does dietary phosphate restriction do more harm than good?

7. Understand the determinants of ectopic calcification and the role played by hyperphosphatemia.

## Figures and Tables

**Table 1.** Prevalence of Identified Complications and Symptoms on Overall, Post-Surgical, Non-Surgical, and Children (<18 years) Patients with Chronic Hypoparathyroidism

	Overall population^a^		Post-surgical adult patients		Non-surgical adult patients		Children patients	
Complications	No. of studies (no. of patients)	Median (%, IQR)	No. of studies (no. of patients)	Median (%, IQR)	No. of studies (no. of patients)	Median (%, IQR)	No. of studies (no. of patients)	Median (%, IQR)
Nephrocalcinosis/nephrolithiasis	55 (13710)	15 (6, 29)	25 (2806)	9 (4, 22)	10 (825)	11 (6, 17)	5(77)	54 (38, 75)
Renal insufficiency	34 (6152)	12 (4, 19)	17 (3633)	10 (4, 16)	7 (744)	13 (8, 36)	3 (57)	0 (0, 21)
Cataract	26 (5463)	17 (9, 44)	10 (2401)	11 (3, 19)	14 (1278)	43 (15,46)	0	-
Seizures	26 (5613)	11 (4, 54)	12 (3268)	5 (2, 9)	11 (1042)	33 (20, 64)	2 (63)	42 (27, 57)
Arrhythmia	10 (3598)	7 (5, 23)	4 (1811)	7 (6, 18)	3 (534)	6 (3, 9)	0	-
Ischemic heart disease	5 (2522)	7 (5, 11)	2 (1374)	8 (5, 11)	3 (534)	7 (3, 19)	0	-
Depression	12 (3107)	9 (3, 19)	5 (1026)	4 (3, 12)	3 (452)	21 (4, 40)	0	-
Infection	13 (11474)	11 (7, 21)	5 (1927)	15 (12, 18)	3 (430)	23 (8, 68)	1 (26)	27
All-cause mortality	11 (2382)	6 (0, 16)	7 (1645)	11 (5, 16)	3 (496)	29 (6, 39)	0	-

Reproduced with permission from Yao and colleagues.^(96)^ IQR = interquartile range.

a Including studies of surgical adult patients, non-surgical adult patients, mixed surgical and non-surgical adult patients, and children patients.

**Table 2.** Characteristics of Studies Investigating PTH Therapy in HypoPT

Study	Country	Study design	Number of patients in PTH/control *n/n)*	Age (mean) (years)	Female (%)	Surgical patients %	Treatment group	Control group	Outcomes	Follow-up duration (months)	Key findings
REPLACE	North America and Europe	Parallel RCT	90/44	47.5	78	74	rhPTH (1–84); active vitamin D; calcium	Placebo; active vitamin D; calcium	Patient important outcomes: adverse events, 50% or greater reduction in dose of vitamin D and calcium, quality of life Surrogate outcomes: hypocalcemia, hypercalcemia, hypercalciuria, 24-hour urine calcium excretion, serum calcium, serum phosphate, serum 25-hydroxyvitamin D, serum 1,25-Dihydroxyvitamin D	6	Serum phosphate levels decreased with rhPTH (1–84). At week 24, serum calcium-phosphate product was lower with rhPTH (1–84) versus placebo. rhPTH (1–84) treatment resulted in significant reductions in oral calcium and active vitamin D dose compared with placebo while maintaining serum calcium. The proportions of patients who had at least one adverse event and serious adverse events were similar between groups.
Sikjaer, Sikjaer, 2011-2014	Denmark	Parallel RCT	32/30	52.0	85	94	rhPTH (1–84); alfacalcidol/calcitriol/ergocalciferol	Placebo; calcium and alfacalcidol/ calcitriol/ergocalciferol	Patient important outcomes: adverse events, quality of life Surrogate outcomes: hypocalcemia, hypercalcemia, 24-hour urine calcium, serum calcium, serum phosphate, serum 25-hydroxyvitamin D, serum 1,25-dihydroxyvitamin D, serum magnesium, Serum alkaline phosphatase, Serum osteocalcin, BMD.	6	Asymptomatic hypercalcemia was present in 71% of the rhPTH (1-84) treated patients. Compared with placebo, 24-hour urinary calcium did not change.
Winer, Winer, 2003	USA	Parallel RCT	14/13	45.0	63	41	PTH (1–34); elemental calcium	Calcitriol and calcium	Patient important outcomes: adverse events Surrogate outcomes: 24-hour urine calcium excretion, serum calcium, serum magnesium, phosphate excretion, mean creatinine clearance, Serum alkaline phosphatase, Serum osteocalcin, Urine deoxypyridinoline, Urine pyridinoline, BMD	36	(1) Serum calcium levels were similar in both treatment groups within or just below the normal range; (2) mean urinary calcium excretion was normal from 1 to 3 years in PTH-treated patients but remained above normal in the calcitriol group; (3) bone mineral content and bone mineral density showed no significant between-group differences over the 3-years
Winer, 1996	USA	Crossover RCT	10	46.5	40	40	PTH (1-34); dietary elemental calcium	Calcitriol; dietary elemental calcium	Patient important outcomes: adverse events Surrogate outcomes: hypercalcemia, 24-hour urine calcium excretion, serum phosphate, serum 25-hydroxyvitamin D, serum 1,25-dihydroxyvitamin D, Serum alkaline phosphatase, Serum osteocalcin, Urine deoxypyridinoline, Urine pyridinoline	2.5	Once-daily PTH (1-34) maintained serum calcium in the normal range with decreased urine calcium excretion compared with calcitriol treatment. Biochemical markers of bone turnover increased significantly during PTH (1-34) treatment.
Winer, 2010	USA	Parallel RCT	7/5	9.6	33	NR	PTH (1-34); dietary elemental calcium; magnesium supplement	Calcitriol; calcium and cholecalciferol; magnesium supplement	Patient important outcomes: adverse events, Surrogate outcomes: hypocalcemia, 24-hour urine calcium excretion, serum calcium, serum phosphate, serum 25-hydroxyvitamin D, serum 1,25-dihydroxyvitamin D, serum magnesium, mean creatinine clearance, Serum alkaline phosphatase, Serum osteocalcin, Urine deoxypyridinoline, Urine pyridinoline, BMD	36	Mean predose serum calcium levels were maintained at, or just below, the normal range, and urine calcium levels remained in the normal range throughout the 3-year study, with no significant differences between treatment groups. Creatinine clearance, did not differ between groups and remained normal throughout the study.
Khan, 2021	North America and Europe	Parallel RCT	44/15	49.2	81	80	TransCon PTH; oral elemental calcium; active vitamin D	Placebo; oral elemental calcium; active vitamin D	Patient important outcomes: adverse events, 50% or greater reduction in dose of vitamin D and calcium, quality of life Surrogate outcomes: hypocalcemia, hypercalcemia, serum calcium, serum phosphate	1	91% of participants treated with TransCon PTH achieved independence from standard of care. Mean 24-h urine Ca decrease by Week 26 while normal serum Ca (sCa) was maintained, and serum phosphate and serum calcium-phosphate product fell within the normal range. TransCon PTH was well tolerated
Khan, 2022	North America and Europe	Parallel RCT	61/21	48.6	78	85	TransCon PTH; oral elemental calcium; active vitamin D	Placebo; oral elemental calcium; active vitamin D	Patient important outcomes: adverse events, quality of life Surrogate outcomes: 24-hour urine calcium excretion, serum calcium, hypocalcemia, hypercalcemia	6.5	93% of participants treated with TransCon PTH achieved independence from conventional therapy. Treatment with TransCon PTH over 26 weeks also normalized mean 24-hour urine calcium. Most adverse events were mild or moderate. No study drug-related withdrawals occurred.

Reproduced with permission from Yao and colleagues.^(6)^

NR = not reported.

**Table 3.** GRADE Summary of Findings

Outcome	Study results and measurements	Absolute effect estimates		Certainty of the evidence (quality of evidence)	Plain text summary
		Conventional therapy^a^	PTH^a^		
Quality of life (physical health)	Measured by: SF-36^b^	−0.1	3.3	Moderate	PTH therapy probably results in a small improvement in quality of life (physical health)
	Scale: 0-100 High better	Mean	Mean	Due to serious imprecision^d^	
	Based on data from 263 patients in 3 studies	Difference: MD 3.4 higher			
	Follow up 6 months	(95% CI 1.5 higher to 5.3 higher)			
Quality of life (mental health)	Measured by: SF-36^b^	−2.8	3	Low	PTH therapy may have small improvement in quality of life (mental health)
	Scale: 0-100 High better	Mean	Mean	Due to very serious imprecision^d^	
	Based on data from 179 patients in 2 studies	Difference: MD 5.8 higher			
	Follow up 5 months	(95% CI 4.9 lower to 16.5 higher)			
Depression	Measured by: WHO-5-well-being	0.1	0.1	Moderate	PTH therapy probably has little or no impact on depression
	Scale: 0–5 High better	Mean	Mean	Due to serious imprecision^e^	
	Based on data from 59 patients in 1 study	Difference: **MD 0 lower**			
	Follow up 6 months	(95% CI 0.2 lower to 0.1 higher)			
50% or greater reduction in doses of active vitamin D and calcium	Relative risk: 6.5	70	455	High	PTH (1-84) and TransCon PTH therapy allow a 50% or greater reduction in doses of active vitamin D and calcium
	(95% CI 2.5–16.4)	per 1000	per 1000		
	Based on data from 191 patients in 2 studies	Difference: 385 more per 1000			
	Follow up 21 months	(95% CI 200 more to 744 more)			
Serious adverse events	Relative risk: 1.1	90	99	Low	PTH therapy may have no or no impact on serious adverse events
	(95% CI 0.6-2.2)	per 1000	per 1000	Due to very serious imprecision^d^	
	Based on data from 349 patients in 5 studies	Difference: 9 more per 1000			
	Follow up 6 months	(95% CI 36 fewer to 108 more)			
Discontinue the study due to adverse events	Relative risk: 1.0	15	15	Low	PTH therapy may have little or no impact on discontinuation due to serious adverse events
	(95% CI 0.1–9.8)	per 1000	per 1000	Due to very serious imprecision^d^	
	Based on data from 216 patients in 2 study	Difference: 0 more per 1000			
	Follow up 6 months	(95% CI 42 fewer to 72 more)			

Reproduced with permission from Yao and colleagues.^(6)^

MID = minimally important difference.

a For continuous outcomes, absolute effect estimates in conventional therapy and PTH groups were difference of baseline and follow up.

b MID is 3 points.^(52)^

c The CI included trivial and small benefits.

d The CI included serious harms and important benefits.

e Small sample sizes.

## Data Availability

The data that support the findings in this study are openly available in PubMed, MEDLINE, EMBASE, and the Cochrane databases.
